# Evaluation of Anti-Inflammatory and Anti-Tubercular Activity of 4-Methyl-7-Substituted Coumarin Hybrids and Their Structure Activity Relationships

**DOI:** 10.3390/ph16091326

**Published:** 2023-09-19

**Authors:** Muthipeedika Nibin Joy, Mallikarjuna R. Guda, Grigory V. Zyryanov

**Affiliations:** 1 Institute of Chemical Engineering, Ural Federal University Named after the First President of Russia B. N. Yeltsin, 28 Mira St., Yekaterinburg 620002, Russia; 2 Ural Division of the Russian Academy of Sciences, I. Ya. Postovskiy Institute of Organic Synthesis, 22 S. Kovalevskoy Street, Yekaterinburg 620219, Russia

**Keywords:** coumarins, anti-inflammatory activity, anti-tubercular activity, SAR

## Abstract

Four sets of previously synthesized 4-methyl-7-substituted coumarin derivatives were screened for their in vitro anti-inflammatory and anti-tubercular activities. The anti-inflammatory potential of **3a**–**t**, **5a**–**o**, **6a**–**n**, and **7a**–**f** synthesized compounds was evaluated by an anti-denaturation assay using diclofenac sodium as the reference standard. Evaluation of the anti-tuberculous activity of the mentioned compounds was performed by the Resazurin test method against four different TB strains using rifampicin and isoniazid as reference drugs. Based on the anti-inflammatory results, compounds **3o**, **5f**, **6c**, and **7d** proved to be the most active compounds in their respective series. Additionally, compounds **3k**–**n**, **5b**–**d**, **6d**–**f**, **6k**, **7a**, and **7f** were found to be the most potent anti-tuberculous agents. In fact, most of the screened compounds exhibited promising activity profiles compared to the respective standard drugs. The structure–activity connections revealed a few intriguing aspects, indicating that the presence of electron-donating and nitrogen-rich fragments boost the anti-inflammatory effects of the examined compounds. However, the presence of electron-withdrawing substituents was required to boost the anti-tubercular activity of the evaluated compounds.

## 1. Introduction

In the modern arena of drug discovery, the role of heterocyclic compounds containing nitrogen and oxygen is increasingly important, as evidenced by their presence in most of the marketed drugs [1,2,3]. Nitrogen- and oxygen-containing heterocycles are also important in the field of material science, agrochemicals, liquid crystals, and fluorescence [4,5,6]. Among the various heterocyclic compounds discovered hitherto, coumarins are a vital class of benzopyrones found in green plants that exhibit profound pharmacological properties [7]. Natural or synthetic coumarins are of tremendous interest to medicinal chemists throughout the globe because of their varied biological activities and potential therapeutic uses [8]. The chemical structure of several marketed drugs like warfarin, acenocoumarol, carbochromen, etc., contains a coumarin nucleus at its core. The various therapeutic applications reported hitherto for coumarin analogs include anti-tumor therapy, photochemotherapy, and anti-HIV therapy [9,10]. The coumarin nucleus is also present in various antibiotics such as clorobiocin, novobiocin, and coumermycin A1 [11,12]. On the other hand, 1,2,3-triazoles are important heterocyclic frameworks found in various bioactive compounds with interesting pharmacological profiles. In addition, the 1,2,3-triazole ring is present in several drug molecules that are available in the market nowadays [13]. 1,2,3-triazoles are successfully used as linkers for various molecules as they possess some important properties, such as high dipole character and hydrogen bonding ability. In addition to this, the 1,2,3-triazole ring is considered a metabolically stable heterocyclic ring due to its high stability under acid/base hydrolysis and oxidative/reductive conditions [14,15]. The various biological properties of compounds containing coumarins and 1,2,3-triazole derivatives include anti-cancer [16], anti-tubercular [17], antimicrobial [18], antiviral [19], antiallergic [20], and anti-inflammatory activities [21]. In view of these observations, the synthesis and exploration of biological activities of novel coumarin and 1,2,3-triazole-based molecules have intrigued chemists for decades in evaluating their applicability as drugs. Accordingly, our efforts were focused on synthesizing new pharmacologically relevant coumarin derivatives and 1,2,3-triazole molecules linked to coumarin.

Generally, inflammation can be termed as a defensive immune response occurring in the body of living organisms against harmful stimuli such as mechanical injury, irritants, or pathogens. Inflammation initiates the healing process of the body by allowing it to undergo various physiological adaptations for reducing tissue damage [22,23]. Conversely, chronic inflammatory conditions can lead to various problems, like autoimmune diseases, organ rejections, and allergies, and are thus considered to be futile for our bodies. Such chronic inflammatory disorders like atherosclerosis, inflammatory bowel diseases, retinitis, psoriasis, multiple sclerosis, osteoarthritis, and rheumatoid arthritis can even lead to mortality [24]. From the aforementioned observations, it is evident that there is a significant need for developing new anti-inflammatory agents all the time. On the other hand, microorganisms like bacteria, fungi, and viruses have been found to pose severe threats to humanity by causing serious health hazards around the globe [25,26]. Regardless of the development of numerous antimicrobial agents, these microorganisms can acquire multi-drug resistance (MDR) which demands the need for identifying new therapeutics to target these pathogens [27]. Therefore, there is a constant requirement to develop new drugs with excellent antimicrobial potency for circumventing this issue. Tuberculosis (TB), a disease caused by bacteria named Mycobacterium tuberculosis, is considered one of the most severe life-threatening diseases worldwide [28]. Even though the main target of this bacterium is the lungs, it can also cause extensive damage to other internal organs. According to the World Health Organization (WHO), until 2021, TB has resulted in more than 1.6 million deaths and 10.6 million clinical cases [29]. Over the past five decades, researchers around the world have been actively working on the development of new anti-TB agents. However, only a few compounds have entered the clinical trials stage. Considering these facts, it is a vital challenge to develop new anti-TB agents with a good mechanism of action, high potency, and low toxicity.

The modern era of drug discovery utilizes the technique of molecular hybridization in developing new heterocyclic frameworks with high pharmacological potency. The molecular hybridization strategy involves the combination of different biologically active heterocyclic moieties into a single entity, thereby leading to compounds with interesting biological profiles. These combined chemical motifs derived from bioactive molecules with different mechanisms of action offer some advantages in overcoming drug resistance and improving the overall pharmacological potency. This is a well-established approach leading to more potent drugs and could be beneficial for the treatment of various diseases in the near future [30,31]. In continuation of our ongoing research work on the synthesis and pharmacological screening of biologically active molecules [32,33], we directed our attention toward evaluating the anti-inflammatory and anti-tubercular properties of some 4-methyl-7-substituted coumarin derivatives. The synthesis of these target compounds was previously reported by our research group [34,35,36]. Accordingly, we herein report the anti-inflammatory and anti-tubercular activity evaluation of these 4-methyl-7-substituted coumarin derivatives. The structure–activity relationship (SAR) studies were also carried out for these previously synthesized compounds at a later stage.

## 2. Results and Discussion

### 2.1. Chemistry

**Scheme 1:**
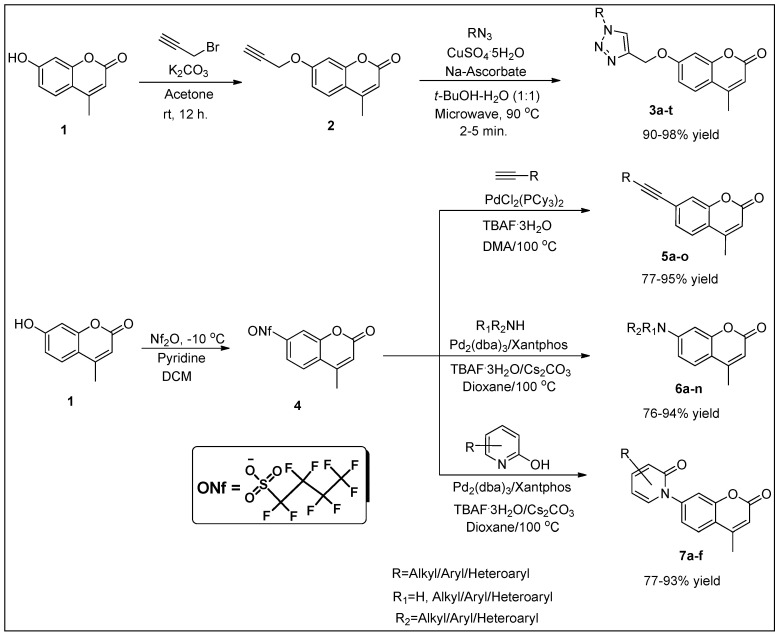
Synthesis of the target compounds.

**Scheme 2:**
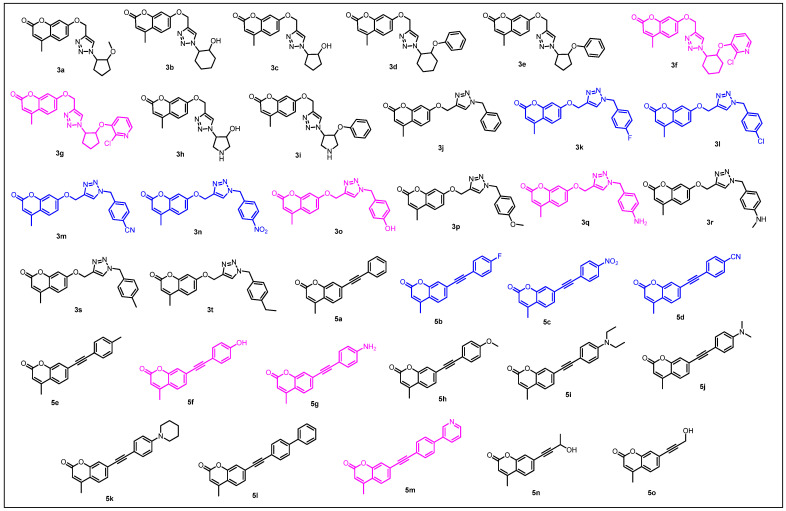
Structure of target compounds **3a**–**t** and **5a**–**o**; the most active anti-inflammatory compounds are in pink, and the most active anti-tubercular agents are in blue.

The synthesis of target compounds **3a**–**t**, **5a**–**o**, **6a**–**n**, and **7a**–**f** has been previously reported by us [34,35,36], as depicted in Scheme 1. Initially, we took 4-methyl-7-hydroxy coumarin **1** as the starting material and treated it with propargyl bromide in K_2_CO_3_ to obtain the O-propargylated product **2**. This alkyne intermediate **2** was then subjected to the copper-catalyzed 1,3 dipolar cycloaddition reaction with various azides under microwave irradiation to synthesize an array of coumarin derivatives linked to 1,2,3-triazole **3a**–**t** (Scheme 2). 

The starting compound **1** was later converted to corresponding nonaflate intermediate **4** by treating it with nonafluorobutanesulfonic anhydride in the presence of pyridine at −10 °C. This intermediate **4** was then subjected to a Sonogashira cross-coupling reaction with the different terminal acetylenes by employing PdCl_2_(PCy_3_)_2_ as a catalyst and TBAF.3H_2_O as a base under microwave irradiation to afford a series of 4-methyl-7-alkynyl coumarins **5a**–**o**. The structures of these target compounds have been illustrated in Scheme 2.

**Scheme 3:**
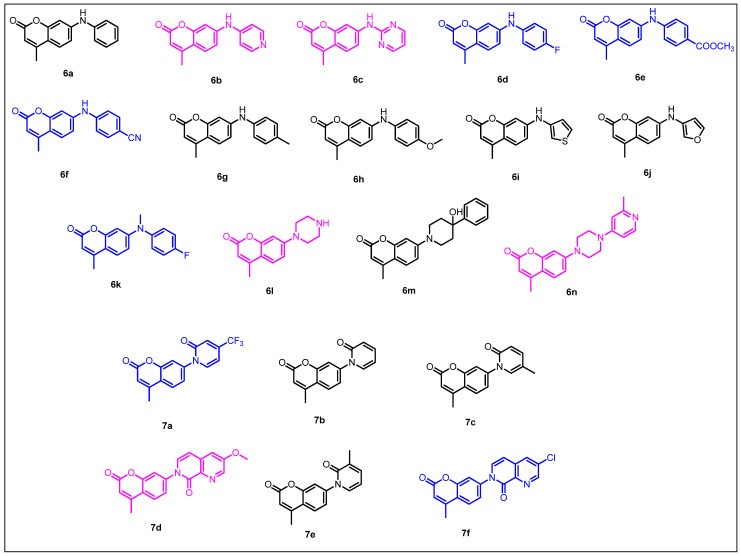
Structure of target compounds **6a**–**n** and **7a**–**f**; the most active anti-inflammatory compounds are in pink, and the most active anti-tubercular agents are in blue.

The intermediate **4** was also subjected to a Buchwald–Hartwig cross-coupling reaction with a variety of amines using a Pd_2_(dba)_3_ catalyst along with a xantphos ligand and TBAF.3H_2_O/Cs_2_CO_3_ as a base under microwave irradiation to furnish an assortment of 4-methyl-7-amino coumarins **6a**–**n**. This methodology was also adopted for synthesizing an array of 4-methyl-7-amido coumarins **7a**–**f** by employing different pyridones as the coupling partner. The structures of these synthesized target compounds have been depicted in Scheme 3. All the target molecules were screened for the evaluation of their in vitro anti-inflammatory and anti-tubercular properties.

### 2.2. The Biological Activity of the Target Compounds

#### 2.2.1. Anti-Inflammatory Activity

All the synthesized target compounds **3a**–**t**, **5a**–**o**, **6a**–**n**, and **7a**–**f** were tested for their in vitro anti-inflammatory activity by an anti-denaturation assay [37]. The starting material **1** was also screened for its anti-inflammatory potential. Diclofenac sodium was used as the reference standard for our screening studies. The synthesized compounds were prepared in various concentrations (100, 200, 400, 800, and 1600 µg/mL) along with the standard and subjected to anti-inflammatory activity evaluation (Table 1).

**Figure 1:**
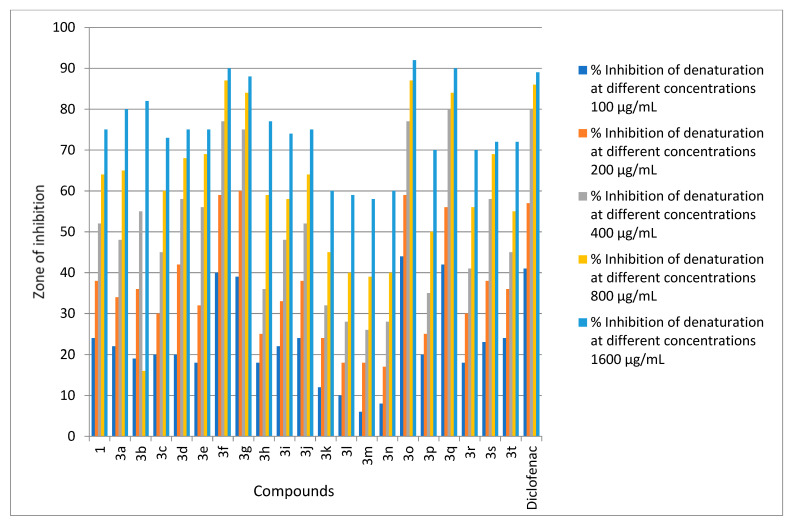
Anti-inflammatory activity of target compounds **3a**–**t** and **1**.

Among the different series of tested compounds, the coumarin linked to 1,2,3-triazole derivatives **3a**–**t** exhibited higher anti-inflammatory activity (6–44% inhibition of denaturation at 100 µg/mL concentration) compared to other molecules. Compound **3o** showed 44% inhibition at 100 µg/mL concentration, whereas diclofenac sodium displayed 41% inhibition at the same concentration. Moreover, compounds **3f**, **3g**, and **3q** exhibited promising anti-inflammatory activity compared to other tested compounds of the same series. These compounds also demonstrated a comparable percentage of inhibition to that of the reference standard at all the other concentrations. On the contrary, compounds **3k**–**n** showed the lowest percentage of inhibition at all the concentrations tested. Therefore, compound **3o** was found to be the most promising one as it displayed a slightly superior activity profile to that of the standard drug, diclofenac sodium (Figure 1). 

**Figure 2:**
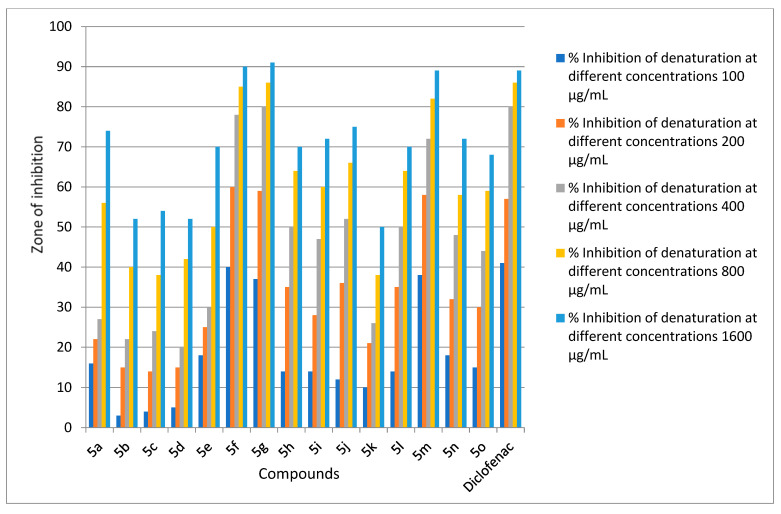
Anti-inflammatory activity of target compounds **5a**–**o**.

4-methyl-7-alkynyl coumarin derivatives **5a**–**o** also displayed promising anti-inflammatory activity compared with the corresponding standard at all the concentrations analyzed (Table 2). Compounds **5f**, **5g**, and **5m** were found to be the most active, as they exhibited a greater percentage inhibition of denaturation at various concentrations. Compounds **5b**–**d** and **5k** showed the lowest activity profile compared to the other evaluated compounds of the same series. All the other tested compounds among these series exhibited moderate to lower activity profiles compared with the standard (Figure 2).

**Figure 3:**
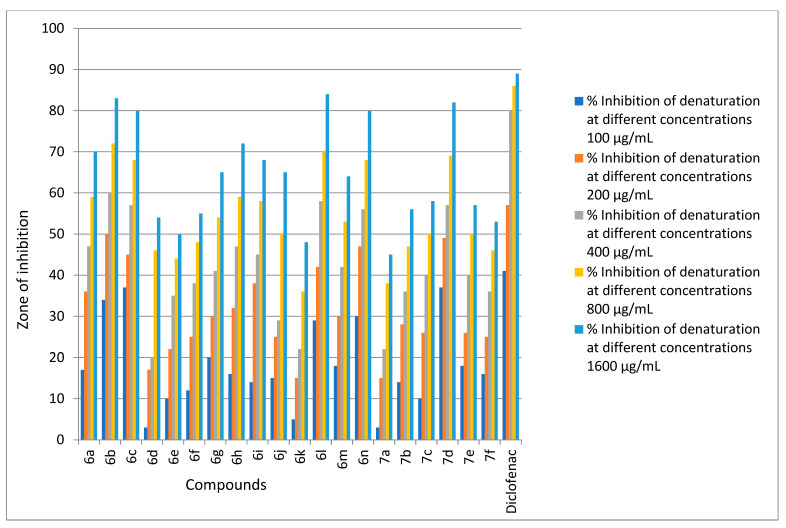
Anti-inflammatory activity of target compounds **6a**–**n** and **7a**–**f**.

Among the 4-methyl-7-amino/amido coumarins **6a**–**n** and **7a**–**f** evaluated for an anti-inflammatory assay, compounds **6b**, **6c**, **6l**, **6n**, and **7d** exhibited superior anti-inflammatory activity (29–37% inhibition of denaturation at 100 µg/mL) compared to other members of this series (Table 3). All the other tested compounds in this series demonstrated a lower activity profile compared with the standard. It is worth noting that the activity profile of this series of molecules was not very promising compared to the reference standard at all the tested concentrations (Figure 3). Hence, it can be concluded that the 4-methyl-7-amino/amido coumarin derivatives have lower anti-inflammatory potential compared to all the other compounds screened.

In summary, the anti-inflammatory potential of all the tested compounds, other than 4-methyl-7-amino/amido coumarins, were found to be promising at different concentrations evaluated. Among the analyzed compounds, **3o**, **5f**, **6c**, and **7d** were found to be the most active compounds in their respective series. However, the compounds containing coumarin linked to 1,2,3-triazole ring (**3a**–**t**) were found to be the most promising anti-inflammatory agents compared to all the other compounds. Furthermore, compound **3o** was found to be the most active, as it exhibited slightly greater potency compared to the reference standard.

#### 2.2.2. Structure–Activity Relationship (SAR) Studies

To identify the correlation between the activity profile and the structural specificity of the tested molecules, we carried out SAR studies. In this work, we have investigated the anti-inflammatory potential of the different series of previously synthesized 4-methyl-7-substituted coumarin analogs. We have screened 55 different compounds based on coumarins containing 1,2,3-triazole ring, alkyne functionality, and amine/amide functional groups. The compounds containing diverse electronic and steric features were included in our anti-inflammatory assay. The anti-inflammatory potential of the tested compounds varied with some specific electronic features.

Among all the tested compounds, the coumarin derivatives linked to 1,2,3-triazoles **3a**–**t** were found to be the most active. This underlines the importance of 1,2,3-triazole functionality in improving the overall anti-inflammatory activity of the compounds under investigation. The nitrogen-enriched triazole nucleus is presumed to enhance the percentage inhibition of denaturation, thereby increasing the anti-inflammatory potential. Among the various compounds screened in this series, compounds **3f**, **3g**, **3o**, and **3q** were identified to be the most potent. The enhanced activity of compounds **3o** and **3q** can be rationalized by the presence of electron-donating -OH and -NH_2_ groups, whereas compounds **3f** and **3g** contain one pyridine fragment, which also has a nitrogen atom. This could be the possible reason for the superior activity profile of **3f** and **3g** compared to all the other tested molecules. On the other hand, the compounds containing electron-withdrawing groups were found to be the least active in this series.

Among the 4-methyl-7-alkynyl coumarin derivatives **5a**–**o**, compounds **5f**, **5g**, and **5m** were found to be the best anti-inflammatory agents. Compounds **5f** and **5g** contain electron-donating -OH and -NH_2_ groups, whereas **5m** possesses a pyridine fragment. On the other hand, the least active compounds **5b**–**d** and **5k** contain electron-withdrawing groups. The lower activity profile of these could be possibly due to the presence of electron-withdrawing substituents. The 4-methyl-7-amino/amido coumarins **6a**–**n** and **7a**–**f** exhibited lower anti-inflammatory activity compared to other tested compounds. However, compounds **6b**, **6c**, **6l**, **6n**, and **7d** exhibited slightly better activity compared to other members of the same series. These compounds contain nitrogen-rich fragments, a possible reason for their optimal activity profile. However, compound **6m**, containing an -OH group, was found to be moderately active only. In general, the compounds containing amide functional groups in coumarins are not found to be promising for our anti-inflammatory potential studies. Therefore, it could be concluded that in the present investigation, the compounds with electron-donating groups and nitrogen-rich fragments exhibit superior anti-inflammatory potential, whereas the compounds containing electron-withdrawing groups show significantly lower anti-inflammatory activity [38].

#### 2.2.3. Anti-Tubercular Activity Studies

Anti-tubercular activity studies of target compounds **3a**–**t**, **5a**–**o**, **6a**–**n**, and **7a**–**f** (along with **1**) were carried out against four different TB strains by the Resazurin assay method using rifampicin and isoniazid as reference standards [39]. Our screening results of the in vitro antimycobacterial activity of the target molecules that are carried out in two stages are summarized in Table 4, Table 5 and Table 6. The anti-tubercular evaluations were primarily carried out at 1, 10, and 100 μg/mL concentrations against three different TB strains. The minimum inhibitory concentration (MIC) values were determined for the tested compounds. In the second stage, the more active molecules from the preliminary tests were further subjected to the next level of screening at lower concentrations. At this phase, the activity against the *MDR-TB* strain was also evaluated along with three previously screened TB strains. Ten μg/mL concentrations were taken as the cut-off at this second phase, and the compounds that were active at 100 μg/mL or more were ignored. At this stage, all the selected compounds were evaluated at 0.3125, 0.625, 1.25, 2.5, and 5.0 μg/mL concentrations.

Among the synthesized target compounds **3a**–**t**, the anti-tubercular activity was found to be promising for some of the molecules, as they were identified to be potent in the range of 1 and 10 μg/mL concentrations against *Mycobacterium tuberculosis* H37Rv strains (Table 4). Among the tested compounds, **3k** and **3m** were identified to be potent at 0.625 μg/mL concentrations against *Mycobacterium tuberculosis* H37Rv strains, and compounds **3l** and **3n** were found to be active at 1.25 μg/mL concentrations. However, compound **3k** showed promising anti-tubercular activity against *Mycobacterium smegmatis* (ATCC 19420) at 1.25 μg/mL. It is noteworthy that most of the tested compounds in this series were active against *Mycobacterium fortuitum* (ATCC 19542) compared with the standard drug, isoniazid. Furthermore, compounds **3k**, **3l**, and **3n** exhibited promising anti-tubercular activity against the *MDR-TB* strain at 6.25 μg/mL. All the other analyzed compounds in this series possessed lower or moderate activity profiles against the tested TB strains.

Among the 4-methyl-7-alkynyl coumarin derivatives **5a**–**o**, compounds **5b**–**d** were found to be the most active (Table 5). All the other tested compounds in this series showed lower or moderate activity profiles against the four TB strains. Compounds **5b**, **5c**, and **5d** were active against *Mycobacterium tuberculosis H37Rv* strains at 0.625, 1.25, and 0.625 μg/mL concentrations, respectively. In addition to this, they were found to be active against *Mycobacterium smegmatis* at 1.25, 5, and 1.25 μg/mL concentrations, respectively. The anti-tubercular activity of **5b** and **5d** were found to be promising against *Mycobacterium fortuitum* compared with the standard drugs, isoniazid and rifampicin. Compounds **5b** and **5c** were found to be potent against the *MDR-TB* strain at 6.25 μg/mL concentrations. 

Among the target compounds **6a**–**n** and **7a**–**f**, the anti-tubercular activity was found to be promising for most of the molecules (Table 6). The 4-methyl-7-amido coumarin derivatives **7a**–**f** (except **7d**) were found to be potent in the range of 1 and 10 μg/mL concentrations against *Mycobacterium tuberculosis H37Rv* strains. Among the tested compounds, **6d**–**f**, **6k**, **7a**, and **7f** were identified to be the most potent ones. Against *Mycobacterium tuberculosis H37Rv* strains, compounds **6d** and **6f** were found to be active at 0.625 μg/mL concentrations, whereas compounds **6e**, **6k**, and **7f** were found to be active at 1.25 μg/mL concentrations. Compounds **6d** and **6f** displayed promising anti-tubercular activity against *Mycobacterium smegmatis* (ATCC 19420) at a concentration of 1.25 μg/mL. However, compounds **6d** and **6f** were active against *Mycobacterium fortuitum* (ATCC 19542) at 5 μg/mL concentrations. Furthermore, compounds **6d**–**f**, **6k**, and **7f** exhibited promising anti-tubercular activity against the *MDR-TB* strain at a concentration of 6.25 μg/mL. All the other tested compounds in this series possessed a moderate activity profile against the four TB strains subjected to evaluation.

#### 2.2.4. SAR Studies

The promising anti-tubercular potential of the 4-methyl-7-substituted coumarin derivatives **3a**–**t**, **5a**–**o**, **6a**–**n**, and **7a**–**f** encouraged us to study their structure–activity relationships. Compounds **7a**–**f**, which contain amide functionality, were found to be active against all the tested TB strains. The amide groups are generally considered good linkers that can effectively bind toward the active site of the proteins [40]. This could be the possible reason for the improved anti-tubercular potential of these 4-methyl-7-amido coumarin derivatives. However, compound **7d** was found to be inactive against all the tested TB strains. Among the other series of target molecules **3a**–**t**, **5a**–**o**, and **6a**–**n**, compounds **3k**–**n**, **5b**–**d**, **6d**–**f**, **6k**, **7a**, and **7f** were found to be the most active. It is noteworthy that all these compounds contain electron-withdrawing substituents such as F, NO_2_, CN, Cl, or CF_3_ attached to the phenyl ring. This could be the major reason for the superior activity profile of these tested compounds. The introduction of electron-withdrawing substitutions at the ring is presumed to increase the lipophilicity of the molecule. This will enhance their cell permeability and enhance the overall potency of these compounds [41].

## 3. Materials and Methods

### 3.1. General Considerations

All chemicals, solvents, reagents, and reference standards were purchased from commercial suppliers and used as delivered. Turbidity was measured using a right-angle scattered light turbidity detector TB820D instrument. The synthesis of target compounds **3a**–**t**, **5a**–**o**, **6a**–**n**, and **7a**–**f** investigated for in vitro anti-inflammatory and anti-tubercular activity in this study has been previously reported by us [34,35,36]. The anti-inflammatory activity of the target compounds was carried out by an anti-denaturation assay by employing diclofenac sodium as the reference standard. Anti-tubercular activity studies were carried out against four different TB strains (*M. tuberculosis*, *M. smegmatis*, *M. fortuitum,* and *MDR-TB* strains) by the Resazurin assay method using rifampicin and isoniazid as reference standards. 

### 3.2. Procedure for Determining In Vitro Anti-Inflammatory Activity: Anti-Denaturation Assay

The experiment was performed according to a previously reported protocol [42]. The extracts of target compounds or drugs were dissolved in a minimum quantity of DMSO and diluted with a phosphate buffer (0.2 M, PH 7.4). It was carefully noted that the final concentration of DMSO in all solutions was less than 2.5%. The test solution (4 mL) containing various concentrations of the target compounds was mixed with 1 mL of a 1 mM solution of albumin in a phosphate buffer and incubated for 15 min at 37 °C. Denaturation was induced by placing the reaction mixture at 70 °C for 15 min in a water bath. The reaction mixture was cooled after 15 min, and the turbidity was measured at 660 nm. A control experiment was also carried out without adding the tested target compounds. Diclofenac sodium was employed as the standard drug for reference. The percentage of inhibition of denaturation was calculated from the control using the following formula:

% of Inhibition = 100 × (At − Ac)/At

At = optical density of test solution; Ac = optical density of control.

### 3.3. Procedure for Determining Anti-Tuberculosis Potential

The in vitro antimycobacterial activity of the target compounds was determined by the Resazurin assay method. The compounds were examined against *M. tuberculosis H37Rv* American Type Culture Collection (ATCC) 27294 and non-tubercular mycobacterial (NTM) species such as *M. smegmatis* (MC2) ATCC 19420, *M. fortuitum* ATCC 19542, and *MDR-TB* strains. The MIC values for each target compound were determined against the tested tubercular strains. The standard drugs used for reference were isoniazid and rifampicin. The *M. tuberculosis* strains were full-grown in Middlebrook 7H9 broth (Difco BBL, Sparks, MD, USA) and supplemented by 10% Oleic Albumin Dextrose Catalase (OADC, Becton Dickinson, Sparks, MD, USA). Using the same medium, the culture was then diluted to McFarland 2 standard. A total of 50 mL of the culture from this standard solution was then added to 150 mL of a fresh medium in 96-well microtitre plates. The test compounds were prepared as stock solutions (2 mg/mL) in N,N-Dimethyl formamide (DMF). Initially, the target compounds compounds were tested at 1, 10, and 100 µg/mL concentrations. Later, the second level of testing was carried out for more active compounds at 0.3125, 0.625, 1.25, 2.5, and 5 µg/mL concentrations. The control tubes were made up of the same volumes of DMF without any substrate. After incubating the stock solution for 7 days at 37 ℃, 20 mL of 0.01% Resazurin (Sigma, St. Louis, MO, USA) in water was added to each tube. Resazurin is a redox dye that is blue in the oxidized state and turns pink when reduced by the growth of viable cells. The control tubes showed a change in color from blue to pink after 1 h at 37 ℃. The test compounds that prevented the color change in the dye were considered to be inhibitory to the tested TB strains. Each experiment was carried out in triplicate.

## 4. Conclusions

In summary, we have successfully determined the evaluation of anti-inflammatory and anti-tubercular activities of 55 previously synthesized 4-methyl-7-substituted coumarin derivatives. The anti-inflammatory potential was evaluated by an anti-denaturation assay using diclofenac sodium as the reference standard, whereas the anti-tubercular activity evaluation of these compounds was carried out by the Resazurin assay method using rifampicin and isoniazid as the reference drugs. Compound **3o** was found to be the most active anti-inflammatory agent, and compounds **3k**, **5b**, **6d**, and **6f** were identified as the most potent anti-tubercular agents. The SAR studies revealed the importance of the presence of electron-donating and nitrogen-rich fragments in improving the anti-inflammatory activities of the compounds investigated. On the other hand, the presence of electron-withdrawing substituents was found to be necessary for enhancing the anti-tubercular activity of the tested compounds.

## Figures and Tables

**Table 1:** Results of the in vitro anti-inflammatory activity studies of compounds **3a**–**t** and **1** ^a^.

Compounds	% Inhibition of Denaturation at Different Concentrations				
	100 µg/mL	200 µg/mL	400 µg/mL	800 µg/mL	1600 µg/mL
**1**	24 ± 0.60	38 ± 0.59	52 ± 1.05	64 ± 0.56	75 ± 0.18
**3a**	22 ± 0.17	34 ± 0.14	48 ± 0.48	65 ± 1.05	80 ± 1.74
**3b**	19 ± 0.45	36 ± 0.14	55 ± 1.17	16 ± 1.14	82 ± 0.75
**3c**	20 ± 0.47	30 ± 1.02	45 ± 0.17	60 ± 0.87	73 ± 0.55
**3d**	20 ± 0.17	42 ± 0.78	58 ± 1.51	68 ± 1.08	75 ± 0.66
**3e**	18 ± 0.11	32 ± 0.78	56 ± 0.15	69 ± 0.63	75 ± 1.03
**3f**	40 ± 0.45	59 ± 0.45	77 ± 1.12	87 ± 0.16	90 ± 0.17
**3g**	39 ± 0.45	60 ± 0.74	75 ± 0.64	84 ± 0.25	88 ± 0.86
**3h**	18 ± 1.12	25 ± 1.01	36 ± 1.12	59 ± 0.23	77 ± 1.12
**3i**	22 ± 0.48	33 ± 0.15	48 ± 0.56	58 ± 0.14	74 ± 0.45
**3j**	24 ± 1.67	38 ± 0.45	52 ± 1.12	64 ± 1.08	75 ± 1.08
**3k**	12 ± 0.56	24 ± 1.03	32 ± 0.05	45 ± 0.56	60 ± 1.12
**3l**	10 ± 0.54	18 ± 0.56	28 ± 1.17	40 ± 1.72	59 ± 1.17
**3m**	6 ± 0.15	18 ± 1.15	26 ± 0.78	39 ± 1.45	58 ± 0.15
**3n**	8 ± 0.46	17 ± 0.18	28 ± 0.05	40 ± 1.03	60 ± 0.05
**3o**	44 ± 1.05	59 ± 1.16	77 ± 0.65	87 ± 0.12	92 ± 0.45
**3p**	20 ± 1.11	25 ± 1.12	35 ± 0.14	50 ± 0.04	70 ± 0.12
**3q**	42 ± 1.05	56 ± 0.17	80 ± 0.65	84 ± 0.02	90 ± 1.01
**3r**	18 ± 0.87	30 ± 0.64	41 ± 1.05	56 ± 0.63	70 ± 1.30
**3s**	23 ± 0.14	38 ± 0.44	58 ± 1.09	69 ± 0.51	72 ± 0.14
**3t**	24 ± 0.56	36 ± 0.15	45 ± 1.54	55 ± 1.14	72 ± 0.17
Diclofenac	41 ± 0.11	57 ± 0.46	80 ± 1.04	86 ± 1.10	89 ± 1.12

^a^ Most active compounds in bold. The experiments were performed in triplicate and expressed as mean ± SD.

**Table 2:** Results of the in vitro anti-inflammatory activity studies of compounds **5a**–**o** ^a^.

Compounds	% Inhibition of Denaturation at Different Concentrations				
	100 µg/mL	200 µg/mL	400 µg/mL	800 µg/mL	1600 µg/mL
**5a**	16 ± 0.55	22 ± 1.55	27 ± 0.87	56 ± 0.48	74 ± 0.60
**5b**	3 ± 0.48	15 ± 0.46	22 ± 0.83	40 ± 1.68	52 ± 1.33
**5c**	4 ± 0.87	14 ± 0.58	24 ± 0.60	38 ± 0.72	54 ± 0.98
**5d**	5 ± 0.57	15 ± 0.47	20 ± 0.41	42 ± 0.97	52 ± 0.32
**5e**	18 ± 1.60	25 ± 0.74	30 ± 0.23	50 ± 1.25	70 ± 0.74
**5f**	40 ± 0.38	60 ± 0.95	78 ± 0.65	85 ± 0.36	90 ± 1.82
**5g**	37 ± 0.45	59 ± 0.87	80 ± 0.79	86 ± 0.77	91 ± 0.36
**5h**	14 ± 1.36	35 ± 1.55	50 ± 0.33	64 ± 0.89	70 ± 0.22
**5i**	14 ± 0.81	28 ± 0.23	47 ± 1.60	60 ± 1.11	72 ± 0.60
**5j**	12 ± 0.90	36 ± 0.54	52 ± 0.67	66 ± 0.49	75 ± 1.62
**5k**	10 ± 0.76	21 ± 0.79	26 ± 0.86	38 ± 0.25	50 ± 0.55
**5l**	14 ± 0.55	35 ± 0.57	50 ± 0.74	64 ± 0.78	70 ± 1.22
**5m**	38 ± 1.05	58 ± 0.69	72 ± 0.36	82 ± 0.93	89 ± 0.63
**5n**	18 ± 0.78	32 ± 1.32	48 ± 0.77	58 ± 0.98	72 ± 0.75
**5o**	15 ± 0.95	30 ± 0.44	44 ± 0.99	59 ± 0.77	68 ± 0.19
Diclofenac	41 ± 0.70	57 ± 0.78	80 ± 1.03	86 ± 0.85	89 ± 0.60

^a^ Most active compounds in bold. The experiments were performed in triplicate and expressed as mean ± SD.

**Table 3:** Results of the in vitro anti-inflammatory activity studies of compounds **6a**–**n** and **7a**–**f** ^a^.

Compounds	% Inhibition of Denaturation at Different Concentrations				
	100 µg/mL	200 µg/mL	400 µg/mL	800 µg/mL	1600 µg/mL
**6a**	17 ± 0.23	36 ± 0.35	47 ± 0.80	59 ± 0.49	70 ± 1.42
**6b**	34 ± 0.57	50 ± 0.87	60 ± 0.63	72 ± 1.71	83 ± 0.29
**6c**	37 ± 0.47	45 ± 1.23	57 ± 0.26	68 ± 0.96	80 ± 0.12
**6d**	3 ± 0.76	17 ± 0.24	20 ± 1.04	46 ± 0.80	54 ± 0.72
**6e**	10 ± 0.88	22 ± 0.92	35 ± 0.65	44 ± 0.36	50 ± 1.23
**6f**	12 ± 0.23	25 ± 0.83	38 ± 0.92	48 ± 0.23	55 ± 0.70
**6g**	20 ± 0.28	30 ± 0.33	41 ± 0.90	54 ± 0.74	65 ± 0.43
**6h**	16 ± 0.64	32 ± 0.42	47 ± 0.25	59 ± 0.93	72 ± 0.64
**6i**	14 ± 0.45	38 ± 0.61	45 ± 0.46	58 ± 1.23	68 ± 0.35
**6j**	15 ± 0.36	25 ± 0.82	29 ± 0.39	50 ± 1.65	65 ± 0.24
**6k**	5 ± 0.97	15 ± 0.61	22 ± 0.28	36 ± 1.01	48 ± 0.20
**6l**	29 ± 0.52	42 ± 0.29	58 ± 0.60	70 ± 0.29	84 ± 1.67
**6m**	18 ± 0.28	30 ± 1.26	42 ± 1.25	53 ± 0.27	64 ± 0.23
**6n**	30 ± 0.82	47 ± 0.23	56 ± 0.28	68 ± 0.35	80 ± 1.39
**7a**	3 ± 1.20	15 ± 0.36	22 ± 0.84	38 ± 0.58	45 ± 0.25
**7b**	14 ± 0.27	28 ± 0.78	36 ± 0.42	47 ± 0.82	56 ± 0.39
**7c**	10 ± 0.50	26 ± 0.86	40 ± 0.97	50 ± 0.09	58 ± 0.65
**7d**	37 ± 0.39	49 ± 0.87	57 ± 1.26	69 ± 0.68	82 ± 0.89
**7e**	18 ± 1.28	26 ± 0.45	40 ± 1.05	50 ± 0.36	57 ± 0.51
**7f**	16 ± 0.24	25 ± 1.28	36 ± 0.69	46 ± 0.47	53 ± 0.45
Diclofenac	41 ± 0.68	57 ± 1.62	80 ± 1.44	86 ± 1.04	89 ± 0.23

^a^ Most active compounds in bold. The experiments were performed in triplicate and expressed as mean ± SD.

**Table 4:** Anti-tubercular activity data of the synthesized compounds **3a**–**t** and **1** ^e^.

Preliminary In Vitro Screening Results, MIC (µg/mL)					Second Level Screening Results, MIC (µg/mL)			
Compound	MTB ^a^	MS ^b^	MF ^c^	% ^d^	MTB	MS	MF	MDR-TB
**1**	10 ± 0.05	10 ± 0.05	>100	<90	>5	>5	>5	>50
**3a**	>100	10 ± 0.05	>100	<90	-	>5	-	25 ± 0.54
**3b**	>100	>100	>100	0	-	-	-	-
**3c**	>100	10 ± 0.07	>100	<90	-	>5	-	25 ± 0.18
**3d**	>100	10 ± 0.07	>100	<90	-	>5	-	25 ± 0.46
**3e**	>100	>100	>100	0	-	-	-	-
**3f**	10 ± 0.10	10 ± 0.20	>100	<90	>5	-	>5	>50
**3g**	10 ± 0.25	10 ± 0.30	>100	<90	>5	-	>5	>50
**3h**	>100	>100	>100	0	-	-	-	-
**3i**	>100	10 ± 0.05	>100	<90	-	>5	-	25 ± 0.28
**3j**	>100	>100	>100	0	-	-	-	-
**3k**	1 ± 0.15	1 ± 0.25	10 ± 0.35	90	0.625 ± 0.07	1.25 ± 0.11	5 ± 0.57	6.25 ± 0.11
**3l**	1 ± 0.19	1 ± 0.30	10 ± 0.25	90	1.25 ± 0.40	5 ± 0.27	>5	6.25 ± 0.12
**3m**	1 ± 0.38	10 ± 0.04	1 ± 0.29	90	0.625 ± 0.34	>5	>5	12.5 ± 0.22
**3n**	1 ± 0.51	1 ± 0.60	10 ± 0.23	90	1.25 ± 0.95	5± 0.33	>5	6.25 ± 0.20
**3o**	>100	>100	>100	0	-	-	-	-
**3p**	10 ± 0.51	10 ± 0.22	>100	<90	>5	-	>5	>50
**3q**	>100	>100	>100	0	-	-	-	-
**3r**	>100	10 ± 0.31	>100	<90	-	-	-	25 ± 0.01
**3s**	10 ± 0.52	10 ± 0.71	>100	<90	>5	-	>5	>50
**3t**	>100	10 ± 0.15	>100	<90	-	-	-	25 ± 0.05
Isoniazid	0.7 ± 0.03	50 ± 0.65	12.5 ± 0.14	95	0.7 ± 0.18	50 ± 0.08	12.5 ± 0.14	12.5 ± 0.09
Rifampicin	0.5 ± 0.65	1.5 ± 0.32	1.5 ± 0.22	95	0.5 ± 0.61	1.5 ± 0.03	1.5 ± 0.28	25 ± 0.11

^a^ *Mycobacterium tuberculosis* H37Rv; ^b^ *Mycobacterium smegmatis* (ATCC 19420); ^c^ *Mycobacterium fortuitum* (ATCC 19542); ^d^ percentage of inhibition against *M. tuberculosis* H37Rv; ‘-’ not detected. ^e^ Most active compounds in bold. The MIC values were performed in triplicate and expressed as mean ± SD.

**Table 5:** Anti-tubercular activity data of the target compounds **5a**–**o** ^e^.

Preliminary In Vitro Screening Results, MIC (µg/mL)					Second Level Screening Results, MIC (µg/mL)			
Compound	MTB ^a^	MS ^b^	MF ^c^	% ^d^	MTB	MS	MF	MDR-TB
**5a**	>100	10 ± 0.15	>100	<90	-	>5	-	25 ± 0.11
**5b**	1 ± 0.45	1 ± 0.75	10 ± 0.42	90	0.625 ± 0.17	1.25 ± 0.38	5 ± 0.51	6.25 ± 0.23
**5c**	10 ± 0.05	10 ± 0.11	10 ± 0.34	90	1.25 ± 0.41	5 ± 0.28	>5	6.25 ± 0.12
**5d**	1 ± 0.17	1 ± 0.22	10 ± 0.41	90	0.625 ± 0.24	>5	5 ± 0.05	12.5 ± 0.21
**5e**	>100	10 ± 0.08	>100	<90	-	>5	-	25 ± 0.05
**5f**	>100	>100	>100	0	-	-	-	-
**5g**	>100	>100	>100	0	-	-	-	-
**5h**	10 ± 0.51	10 ± 0.15	>100	<90	>5	-	>5	>50
**5i**	10 ± 0.28	10 ± 0.18	>100	0	>5	-	>5	>50
**5j**	10 ± 0.14	10 ± 0.85	>100	<90	>5	-	-	>50
**5k**	10 ± 0.16	10 ± 0.39	>100	<90	>5	>5	>5	25 ± 0.48
**5l**	10 ± 0.33	10 ± 0.28	>100	<90	>5	-	>5	>50
**5m**	>100	>100	>100	0	-	-	-	-
**5n**	10 ± 0.20	10 ± 0.65	>100	<90	>5	-	>5	>50
**5o**	>100	10 ± 0.21	>100	<90	-	>5	-	25 ± 0.61
Isoniazid	0.7 ± 0.10	50 ± 0.09	12.5 ± 0.34	95	0.7 ± 0.05	50 ± 0.01	12.5 ± 0.16	12.5 ± 0.38
Rifampicin	0.5 ± 0.17	1.5 ± 0.14	1.5 ± 0.11	95	0.5 ± 0.84	1.5 ± 0.54	1.5 ± 0.13	25 ± 0.74

^a^ *Mycobacterium tuberculosis* H37Rv; ^b^ *Mycobacterium smegmatis* (ATCC 19420); ^c^ *Mycobacterium fortuitum* (ATCC 19542); ^d^ percentage of inhibition against *M. tuberculosis* H37Rv; ‘-‘ not detected. ^e^ Most active compounds in bold. The MIC values were performed in triplicate and expressed as mean ± SD.

**Table 6:** Anti-tubercular activity data of target compounds **6a**–**n** and **7a**–**f** ^e^.

Preliminary In Vitro Screening Results, MIC (µg/mL)					Second Level Screening Results, MIC (µg/mL)			
Compound	MTB ^a^	MS ^b^	MF ^c^	% ^d^	MTB	MS	MF	MDR-TB
**6a**	10 ± 0.19	10 ± 0.17	>100	<90	>5	-	>5	>50
**6b**	10 ± 0.45	10 ± 0.11	>100	<90	>5	-	>5	>50
**6c**	>100	>100	>100	0	-	-	-	-
**6d**	1 ± 0.50	1 ± 0.45	10 ± 0.31	90	0.625 ± 0.18	1.25 ± 0.74	5 ± 0.14	6.25 ± 0.26
**6e**	1 ± 0.61	1 ± 0.10	10 ± 0.34	90	1.25 ± 0.41	5 ± 0.07	>5	6.25 ± 0.16
**6f**	1 ± 0.28	1 ± 0.07	10 ± 0.31	90	0.625 ± 0.23	1.25 ± 0.14	5 ± 0.05	6.25 ± 0.22
**6g**	10 ± 0.15	10 ± 0.70	>100	<90	>5	-	>5	>50
**6h**	>100	>100	>100	0	-	-	-	-
**6i**	>100	10 ± 0.04	>100	<90	-	>5	-	25 ± 0.41
**6j**	>100	>100	>100	0	-	-	-	-
**6k**	1 ± 0.21	1 ± 0.44	10 ± 0.13	90	1.25 ± 0.02	5 ± 0.21	>5	6.25 ± 0.56
**6l**	>100	>100	>100	0	-	-	-	-
**6m**	>100	>100	>100	0	-	-	-	-
**6n**	>100	10 ± 0.09	>100	<90	-	>5	-	25 ± 0.54
**7a**	10 ± 0.18	10 ± 0.21	>100	<90	>5	>5	>5	>50
**7b**	10 ± 0.33	10 ± 0.08	>100	<90	>5	-	>5	>50
**7c**	>100	10 ± 0.17	>100	<90	-	>5	-	25 ± 0.54
**7d**	>100	>100	>100	0	-	-	-	-
**7e**	10 ± 0.11	10 ± 0.51	>100	<90	>5	>5	>5	>50
**7f**	1 ± 0.37	1 ± 0.71	10 ± 0.15	90	1.25 ± 0.11	5 ± 0.31	>5	6.25 ± 0.26
Isoniazid	0.7 ± 0.28	50 ± 0.04	12.5 ± 0.17	95	0.7 ± 0.16	50 ± 0.16	12.5 ± 0.05	12.5 ± 0.19
Rifampicin	0.5 ± 0.35	1.5 ± 0.18	1.5 ± 0.16	95	0.5 ± 0.28	1.5 ± 0.64	1.5 ± 0.49	25 ± 0.14

^a^ *Mycobacterium tuberculosis* H37Rv; ^b^ *Mycobacterium smegmatis* (ATCC 19420); ^c^ *Mycobacterium fortuitum* (ATCC 19542); ^d^ percentage of inhibition against *M. tuberculosis* H37Rv; ‘-‘ not detected. ^e^ Most active compounds in bold. The MIC values were performed in triplicate and expressed as mean ± SD.

## Data Availability

Data is contained within the article.
